# Mapping of susceptible variants for cold medicine-related Stevens–Johnson syndrome by whole-genome resequencing

**DOI:** 10.1038/s41525-021-00171-2

**Published:** 2021-02-11

**Authors:** Yosuke Kawai, Yuki Hitomi, Mayumi Ueta, Seik-Soon Khor, Ken Nakatani, Chie Sotozono, Shigeru Kinoshita, Masao Nagasaki, Katsushi Tokunaga

**Affiliations:** 1 Department of Human Genetics, Graduate School of Medicine, The University of Tokyo, Tokyo, Japan; 2 Department of Frontier Medical Science and Technology for Ophthalmology, Kyoto Prefectural University of Medicine, Kyoto, Japan; 3 Department of Ophthalmology, Kyoto Prefectural University of Medicine, Kyoto, Japan; 4 Department of Integrative Genomics, Tohoku Medical Megabank Organization, Tohoku University, Sendai, Japan; 5 Present Address: Genome Medical Science Project (Toyama), National Center for Global Health and Medicine, Tokyo, Japan; 6 Present Address: Department of Microbiology, Hoshi University School of Pharmacy and Pharmaceutical Sciences, Tokyo, Japan; 7 Present Address: Human Biosciences Unit for the Top Global Course Center for the Promotion of Interdisciplinary Education and Research, Kyoto University, Kyoto, Japan; 8 Present Address: Center for Genomic Medicine, Graduate School of Medicine, Kyoto University, Kyoto, Japan

**Keywords:** Disease genetics, Genome-wide association studies, Acute inflammation

## Abstract

Stevens–Johnson syndrome (SJS) and its severe condition with extensive skin detachment and a poor prognosis, toxic epidermal necrolysis (TEN), are immunologically mediated severe cutaneous reactions of the skin and mucous membranes such as the ocular surface. Genetic variations on the *HLA-A* and other autosomal genes have been identified as risk factors for cold medicine-related SJS/TEN with severe ocular complications (CM-SJS/TEN with SOC). Using a whole-genome sequencing (WGS) approach, we explored other susceptible variants of CM-SJS/TEN with SOC, especially among rare variants and structural variants (SVs). WGS was performed on samples from 133 patients with CM-SJS/TEN with SOC and 418 healthy controls to obtain single nucleotide polymorphisms (SNPs) and SVs. Genome-wide association tests were performed with these variants. Our genome-wide association test reproduced the associations of the common variants of *HLA-A* and loci on chromosome 16q12.1. We also identified novel associations of SVs on these loci and an aggregation of rare coding variants on the *TPRM8* gene. In silico gene expression analysis on the *HLA-A* locus revealed that the SNP (rs12202296), which was significantly associated with susceptibility to CM-SJS/TEN with SOC, was correlated to an increase in *HLA-A* expression levels in the whole blood (*P* = 2.9 × 10^−17^), from the GTEx database. The majority of variants that were significantly associated with CM-SJS/TEN with SOC were found in non-coding regions, indicating the regulatory role of genetic variations in the pathogenesis of CM-SJS/TEN with SOC.

## Introduction

Stevens–Johnson syndrome (SJS) and its severe condition with extensive skin detachment and a poor prognosis, toxic epidermal necrolysis (TEN) are immunologically mediated acute inflammatory vesiculobullous reactions on the skin and mucous membranes. It manifests as painful blistering skin rashes, fever, and hematologic abnormalities. Many drugs and infectious agents have been reported to trigger these reactions^1–3^. Although the annual incidence is only 1–6 cases/million, these reactions show high mortality rates (SJS: 3%; TEN 27%)^1–4^. Among those who survive, about 40% have been reported to develop severe ocular complications (SOC)^4,5^. Carbamazepine, which is used primarily for treating epilepsy and neuropathic pain, allopurinol, which is used to decrease blood uric acid levels, and cold medicines (CM), including multi-ingredient cold medications and nonsteroidal anti-inflammatory drugs (NSAIDs), have been implicated in the development of SJS/TEN^4^.

Human leukocyte antigen (HLA) class I genes (*HLA-A*, *HLA-B*, and *HLA-C*) are well-known susceptibility genes for SJS/TEN. HLA-B*15:02 has been shown to be significantly associated with susceptibility to carbamazepine-induced SJS/TEN in the Taiwanese Han Chinese population (odds ratio [OR] = 2,504, *P* = 3.1 × 10^−27^)^6^. HLA-A*31:01 has also been reported to be a carbamazepine-induced SJS/TEN-susceptibility *HLA* allele in Japanese and European populations (OR = 9.5, *P* = 1.1 × 10^−16^ and OR = 15.0, *P* = 3.5 × 10^−8^; respectively)^7,8^. HLA-B*58:01 was also significantly associated with susceptibility to allopurinol-induced SJS/TEN in the Han Chinese, European, and Japanese populations (OR = 580, *P* = 4.7 × 10^−24^; OR = 80, *P* < 10^−6^; and OR = 62.8, *P* = 5.4 × 10^−12^; respectively)^9–11^. Further, HLA-A*02:06 and HLA-B*44:03 were reported as CM-SJS/TEN with SOC-susceptibility *HLA* alleles in the Japanese population (OR = 5.6, *P* = 2.7 × 10^−20^ and OR = 2.0, *P* = 1.3 × 10^−3^; respectively)^12^. We recently performed the high-resolution next-generation sequencing (NGS)-based HLA typing of HLA class I genes in order to test the association of CM-SJS/TEN with 3-field level HLA allele. In this study, we found HLA-A*02:06:01 was strongly associated with susceptibility to CM-SJS/TEN (OR = 5.46, *p* = 1.15 × 10^−18^)^13^. In addition, recent genome-wide association studies (GWAS) have identified *Ikaros Family Zinc Finger 1 (IKZF1)*, chromosome 16q12.1, and chromosome 15q24.1 as non-*HLA* gene loci associated with susceptibility to CM-SJS/TEN in the Japanese population^14,15^. However, because these known genetic factors cannot fully explain the susceptibility of CM-SJS/TEN with SOC, we postulate that other genetic factors affecting the development of CM-SJS/TEN with SOC still remain unidentified.

We have discovered loci associated with susceptibility to CM-SJS/TEN with SOC among SNPs that were directly genotyped by genome-wide SNP arrays^14^ or obtained by genotype imputation^15^. Because genotype imputation can infer virtually all genotypes of common SNPs (minor allele frequency > 5%)^16^, the vast majority of common SNPs among the samples have been tested for association with CM-SJS/TEN with SOC in the previous studies. However, we have not tested the rare SNPs and structural variants (SVs) that could not be analyzed by SNP array genotyping. These variants may affect the susceptibility of the individual to develop complex traits and diseases. Indeed, the functional impact of structural variations is known to be higher than that of single nucleotide alterations^17^. Further, a recently constructed comprehensive map of gene expression has shown that the contribution of SVs to gene expression is not negligible^18^. In this study, we performed a comprehensive analysis of variations by whole-genome sequencing (WGS) of patients with CM-SJS/TEN with SOC to investigate the impact of rare variants and SVs on the development of this disease.

## Results

### SNP and SV discovery from WGS data

We performed WGS of 133 patients with CM-SJS/TEN with SOC and 418 control individuals. Manipulation of DNA library and sequencing of all samples were performed in a single facility. Average read depths of autosomal chromosomes were 41.6 and 41.4 for case and control samples, respectively. We performed the discovery and genotyping of polymorphic variants using four different tools (Supplementary Table 1). After applying quality control (QC), we identified 21,207,465 variants. The most common form of variants was SNV (18,824,284). In addition, we detected various forms of structural variations (SVs): short insertion and deletions (INDEL), large deletions (DEL), duplications (DUP), inversions (INV), mobile element insertions (MEI), and short tandem repeats (STR). INDELs (1,702,192) were the most prevalent SVs, followed by STRs (670,775), MEIs (7821), DELs (2065), DUPs (273), and INVs (55). The number of variants per individual was uniform across case and control samples. In addition, no significant population stratification was observed between case and control samples by the principal component analysis (Supplementary Fig. 1).

### Single variant test

**Fig. 1:**
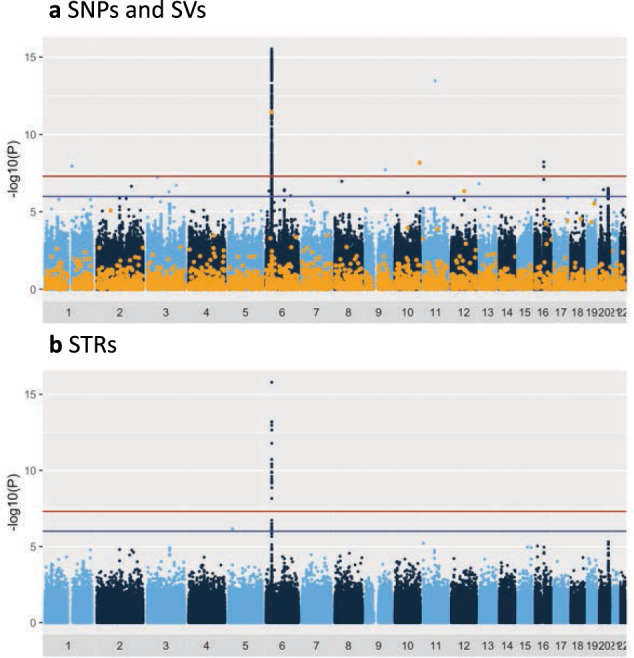
Genome-wide association plot of samples from patients with CM-SJS/TEN with SOC. The negative logarithm of the p values of each variant (Y-axis) were plotted against genomic coordinates on each autosome (X-axis). Red and blue horizontal lines represent *p* values with 5 × 10^−8^ and 1 × 10^−6^, respectively. **a** SNVs are colored dark blue and light blue, while SVs (INDELs, DELs, DUPs, MEIs, and INVs) are colored orange. **b** STRs are colored dark blue and light blue.

**Fig. 2:**
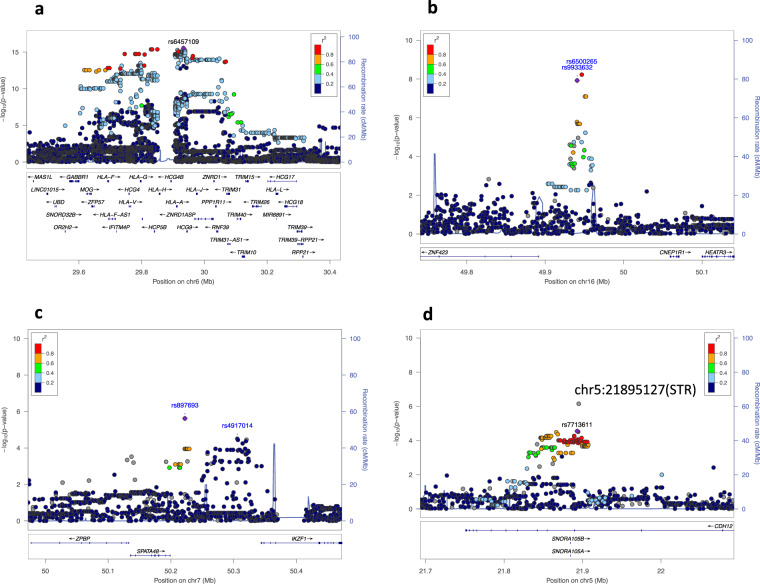
Regional plots for loci with significant associations. Each panel represents the association of variants on four loci: **a** *HLA-A* gene, **b** chromosome 16q12.1, **c** *IKZF1* gene, and **d** chromosome 5p14.3. Each variant is colored according to the strength of LD (r^2^) with top hit variants, except for STRs, which are colored gray.

Each variant was tested for association with the development of CM-SJS/TEN with SOC. After filtering out variants that showed low frequency (minor allele frequency < 1%) and those that departed from Hardy-Weinberg equilibrium (*p* value < 1 × 10^−5^), a total of 7,608,150 variants (6,590,396 SNPs, 579,793 INDELs, 1195 DELs, 73 DUPs, 13 INVs, 2390 MEIs, and 434,290 STRs) were used for the genome-wide association test. Figure 1 shows the genome-wide view of the association test results for SNVs, SVs, and STRs; variants with *p* < 1.0 × 10^−6^ are listed in Supplementary Data 1. Strong associations were aggregated on HLA class I loci (Fig. 2a). For example, the SNP that showed the strongest association (rs6457109) was at the HLA locus (OR = 4.52, *p* = 2.95 × 10^−16^), and an MEI variant, an ALU insertion upstream of *the HLA-*A gene (chr6:29818872) also showed strong association (OR = 2.72, *p* = 3.63 × 10^−12^). The single variant test revealed an association at chromosome 16q12.1 (Fig. 2b). Two SNPs, rs9933632 (OR = 2.60, *p* = 1.19 × 10^−8^) and rs6500265 (OR = 2.65, *p* = 5.96 × 10^−9^), also showed association with genome-wide significance (*p* < 5.0 × 10^−8^) in this region. These SNPs have been previously reported in a GWAS, in which whole-genome imputation was employed^15^. In fact, the effect size and direction of effect of these SNPs were consistent with this study; rs9933632 (OR = 2.28, *p* = 2.38 × 10^−8^) and rs6500265 (OR = 2.33, *p* = 1.16 × 10^−8^)^15^. We also confirmed the known associations of rs897693 (OR = 4.39, *p* = 2.74 × 10^−6^) and rs4917014 (OR = 0.54, *p* = 2.08 × 10^−5^) (Fig. 2c), which are also associated with differential splicing isoforms of *IKZF1*^14^. For STR loci, 434,290 variants were tested after filtration. In addition to the genome-wide significant association around the *HLA-A* gene, we observed a significant STR variant at chromosome 5p14.3 (*p* = 8.31 × 10^−7^) (Fig. 2d). This STR comprises four alleles that have 12, 13, 14, and 15 adenine repeats in the intronic region of the cadherin 12 (*CDH12*) gene (Supplementary Fig. 2). The 12- and 14-repeat alleles exhibited significant associations with *p* values of 5.66 × 10^−5^ and 3.40 × 10^−5^, respectively, while the 15-repeat allele was marginally significant (*p* = 0.003) (Table 1).

**Table 1:** Association of single nucleotide repeats on *CDH12* gene.

Repeat number (allele)	Number of alleles (case)	Number of alleles (control)	Allele frequency (case)	Allele frequency (control)	Odds ratio (95%CI)	*p* value
12	51	83	0.196	0.100	2.19 (1.46–3.25)	8.66 × 10^−5^
13	14	39	0.054	0.047	1.15 (0.57–2.21)	6.24 × 10^−1^
14	191	706	0.735	0.853	0.48 (0.34–0.68)	3.40 × 10^−5^
15	4	0	0.015	0.000	–	3.20 × 10^−3^

### Gene-based test for rare variants

Accumulation of rare (MAF < 1%) protein-altering mutations was assessed by Wald’s test and SKAT-O test. A total of 17,315 protein-coding genes with at least one rare variant were assessed in this analysis. Although no gene exhibited significant association after Bonferroni correction (*p* < 0.05/17315 = 2.89 × 10^−6^), the association shown by one and four genes were suggestive (*p* < 5.77 × 10^−5^), according to the results of the Wald’s test and SKAT-O test, respectively (Table 2). The transient receptor potential cation channel subfamily M member 8 (*TRPM8*) gene was significant in both tests. Among 11 missense single nucleotide variants (SNVs) and two stop-gained SNVs detected in *TRPM8*, eight were detected in CM-SJS/TEN with SOC patients, while two were detected in control samples and three were shared between case and control samples (Supplementary Data 2).

**Table 2:** Gene-based rare variant association test.

Locus	Gene	P value	Test
2q37.1	*TRPM8*	4.96 × 10^−5^	Wald’s test
2q37.1	*TRPM8*	6.85 × 10^−6^	SKAT-O
10p11.21	*PARD3*	1.18 × 10^−5^	SKAT-O
13q12.2	*FLT3*	3.25 × 10^−5^	SKAT-O
2p13.1	*TTC31*	4.62 × 10^−5^	SKAT-O

### The effects of CM-SJS/TEN with SOC-susceptibility polymorphisms in *HLA-A* and chromosome 16q12.1 on gene expression levels

**Fig. 3:**
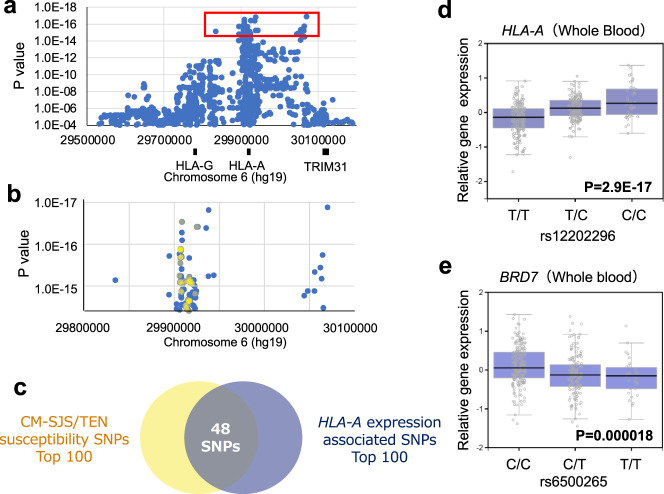
CM-SJS/TEN with SOC-susceptibility polymorphisms in *HLA-A* and chromosome 16q12.1 are associated with gene expression levels in a *cis* manner. **a** The association of polymorphisms in the *HLA-A* locus with the endogenous expression levels of *HLA-A*. Blue dots inside the red rectangle show the 100 SNPs with the strongest association with endogenous expression levels of *HLA-A* in whole blood. **b**, **c** Partial coincidence between the 100 SNPs with the strongest association with susceptibility to CM-SJS/TEN with SOC (dots shown in yellow) and that with endogenous expression levels of *HLA-A* in whole blood (dots shown in blue). **d** Significant association between rs12202296 and the expression level of *HLA-A* in whole blood. CM-SJS/TEN with SOC-susceptibility allele rs12202296 is denoted as the C allele. **e** Significant association between rs6500265 and the expression level of *BRD7* in whole blood. CM-SJS/TEN with SOC-susceptibility allele rs6500265 is denoted as the T allele.

The endogenous expression level of *HLA-A* was strongly associated with polymorphisms in the *HLA-A* locus in a *cis* manner (Fig. 3a). These associations were probably caused by linkage disequilibrium (LD) with primary functional variants that regulated the efficiency *of HLA-A* expression levels. To assess the regulatory effects of *HLA-A* polymorphisms significantly associated with CM-SJS/TEN-susceptibility on gene expression, the differences in *HLA-A* expression levels in whole blood were compared among these SNPs using the GTEx portal database (V7)^19^. Among the SNPs which showed the top-100 of the strongest associations with susceptibility to CM-SJS/TEN with SOC, 48 SNVs were included in the SNPs which showed the top-100 of the strongest associations with the allelic difference of *HLA-A* expressions (Fig. 3b, c). Among these SNVs, the disease susceptibility allele of rs12202296, which showed both significant association with susceptibility to CM-SJS/TEN with SOC and endogenous expression levels of *HLA-A* in whole blood, was associated with an increase in *HLA-A* expression levels (*P* = 2.9 × 10^−17^, Fig. 3d). These results indicated that the significant association of the *HLA-A* locus with susceptibility to CM-SJS/TEN with SOC was caused by the regulatory effects of primary functional polymorphisms (including SNVs, INDELs, and SVs) located near *HLA-A*; further, the expression level of *HLA-A* was up-regulated by the disease susceptibility-associated *HLA-A* polymorphism. We explored the functional effects of polymorphisms that were significantly associated with susceptibility to CM-SJS/TEN with SOC in chromosome 16q12.1 (Fig. 2b). The association between allelic differences of the expression levels of all genes expressed in the whole blood and the SNPs associated with susceptibility to CM-SJS/TEN with SOC were assessed using the GTEx portal database^19^. The disease susceptibility allele rs6500265, one of the SNPs most strongly associated with CM-SJS/TEN with SOC susceptibility, was also associated with a decrease in the expression level of bromodomain-containing 7 *(BRD7)* (*P* = 0.000018, Fig. 3e).

## Discussion

Previous studies on CM-SJS/TEN identified common SNPs and HLA alleles as susceptibility polymorphisms. In this study, we investigated the role of rare and structural variants in the development of CM-SJS/TEN with SOC. So far, the association of these variants with disease susceptibility has been difficult to assess due to technical limitations of SNP array-based GWAS. Therefore, we performed WGS on 133 patients and 418 control subjects, who were previously analyzed by SNP arrays. Extensive variant discovery, followed by joint genotyping of case and control samples, enabled us to test the associations of SNPs and SVs in a genome-wide manner. Unlike other genotyping methods such as SNP array or *HLA* allele typing, WGS allows the evaluation of the disease susceptibilities of all polymorphisms in the human genome. This allows the direct construction of a SNP catalog on both disease susceptibility and allelic differences in gene expressions by combining the WGS data with data from the eQTL database; it also helps us understand the functional mechanisms underlying disease susceptibility. In this study, we used this in silico fine-mapping approach to explore the regulatory role of gene expression in *HLA-A* and *BRD7* loci (Fig. 3). However, it is difficult to adopt the study design in which a large number of samples are enrolled to gain enough statistical power in the GWAS of rare diseases like SJS. Therefore, we could not repeat the association test with replication cohorts. In the future, it is necessary to perform genome-wide metanalysis between replication cohorts from different populations to reinforce the associations found in this study. WGS has the advantage of being able to analyze almost all variants, but it is necessary to consider the increase in potential type 1 errors. This study uses a stringent 5 × 10^−8^ as the genome-wide significant *p* value, but there remains the potential of false positives and false negative findings.

*HLA* class I genes, including *HLA-A*, *HLA-B*, and *HLA-C*, have been reported as the loci most strongly associated with susceptibility to all types of SJS/TEN, including CM-SJS/TEN with SOC. Although non-synonymous substitutions that influence the peptide binding or conformation of HLA molecules have been considered the major factors in the pathogenesis of immunological diseases, our results show that the expression levels of *HLA* genes also play an equally critical role. Indeed, differential expression levels of *HLA-C* in different alleles have been reported, with higher *HLA-C* expression causing an enhanced response of Tc and a deleterious effect in Crohn’s disease^20^. In addition, the expression level of *HLA-A* has been reported to be regulated in an allele-specific manner because of methylation at several CpG sites, depending on *HLA-A* alleles^21^. In the present study, we constructed a SNP catalog on both CM-SJS/TEN with SOC susceptibility and allelic differences in gene expression levels by combining WGS and eQTL and elucidated the effects of SNPs associated with susceptibility to CM-SJS/TEN with SOC on *HLA-A* gene expression levels (Fig. 3b, c). Among these SNPs, the alleles associated with susceptibility to CM-SJS/TEN with SOC showed high *HLA-A* expression levels (Fig. 3d); thus, these alleles probably enhance the response of Tc as well as *HLA-C*^20^.

The association of SNPs with expression levels of *HLA-A* is likely caused by linkage disequilibrium with primary functional SVs that directly regulate *HLA-A* expression. For identifying primary functional polymorphisms, including SNPs and SVs, future studies could compare the *HLA-A* expression levels in cells that are knocked-in with different candidate functional alleles using gene-editing technologies such as the CRISPR/Cas9 system. We had recently used a similar approach to identify primary functional variants in the primary biliary cholangitis (PBC)-susceptibility gene loci *NFKB1/MANBA*^22^.

We had previously used GWAS to confirm that chromosome 16q12.1 was associated with susceptibility to CM-SJS/TEN with SOC^15^. In the present study, we constructed a SNP catalog of chromosome 16q12.1 on both CM-SJS/TEN with SOC susceptibility and allelic differences in gene expression levels by combining WGS and eQTL analysis. We found that the gene *BRD7* showed a significant association between gene expression level and rs6500265, a SNP that was one of the most strongly associated with susceptibility to CM-SJS/TEN with SOC. BRD7 is a cell growth inhibitor that causes cell cycle arrest and apoptosis by directly regulating *BIRC2*, *BIRC3*, *TXN2*, and *NOTCH1*^23–25^. A recent study had reported that BRD7 could repress the Ras/Raf/MEK/ERK pathway and inflammatory cytokines such as interleukin 6 (IL-6) and tumor necrosis factor α (TNFα)^26,27^. In the present study, *BRD7* expression levels were lower in samples with allele of rs6500265, a CM-SJS/TEN-susceptibility SNP in chromosome 16q12.1 (Fig. 3e), according to the GTEx database. Lower expression levels of *BRD7* could possibly attenuate the anti-inflammatory effects and may induce the explosion of inflammatory cytokines in patients with CM-SJS/TEN with SOC.

We successfully reproduced the CM-SJS/TEN with SOC variants that have been previously reported in the Japanese population. Two loci (*HLA-A* and chromosome 16q12.1) exhibiting genome-wide significance (*p* < 5 × 10^−8^) have been previously reported as associated loci of CM-SJS/TEN with SOC. In this study, the most significant association of rs9933632 at 16q12.1 was obtained by direct SNP calling from WGS analysis; we have also reported the association of this SNP by whole-genome imputation analysis^15^. This suggested that whole-genome imputation could cover a vast majority of common variants present in a reference panel, if a haplotype reference panel was matched to the study population^16^. In this study, we focused on SVs and rare variants that could not be imputed due to the limitations of the reference panel. In addition to the common SNPs in genes that have been reported to be associated with susceptibility to CM-SJS/TEN with SOC, novel candidate susceptibility variants were identified among SVs and rare variants. Cadherin 12 (*CDH12*) is a member of the cadherin gene family involved in calcium-dependent homophilic cell–cell adhesion^28^. In the *CDH12* locus, an STR variant exhibited the strongest association with susceptibility to CM-SJS/TEN with SOC (Fig. 2d). Although the contributions of this molecule to immunological reactions and inflammations is unknown, *CDH12* is known to affect vascular permeability in response to inflammation. Further research should be performed to elucidate the molecular mechanisms of disease onset attributed to the STR in the *CDH12* locus, which showed suggestive association with susceptibility to CM-SJS/TEN with SOC in the present study. The rare variant association test identified the aggregation of rare variants that alter the peptide sequences of gene products (Table 2). The gene transient receptor potential melastatin 8 *(TRPM8)* encodes an ion channel that acts as a cold receptor involved in thermosensation^29,30^. The activation of *TRPM8* has also been reported to inhibit UV-B-induced production of prostaglandin E_2_ (PGE_2_), an inflammatory cytokine, in keratinocytes^31^. This result indicated that *TRPM8* was related to inflammatory responses in the skin. The expression of EP_3_, an inhibitory receptor of PGE_2_, was found to be significantly reduced in the conjunctival epithelium on the ocular surface of patients with SJS/TEN with SOC^32^. Similar to such previous reports, rare variants whose allele frequencies are increased in SJS/TEN patients in this study might also modify the function of *TRPM8* and might have effects on the production of PGE_2_. PARD3 (also known as Par3) is an adapter protein localized in the epithelial tight junction; it plays a critical role in it by forming a complex with PARD6 (also known as Par6) and atypical protein kinase C (aPKC)^33,34^. There is evidence that PARD3 contributes to the acceleration of vascular permeability in response to inflammation. Vascular endothelial (VE)-cadherin, Pals1, and PARD3 regulates endothelial polarity and vascular lumen formation^35^. Additionally, in an in vitro study using human monocytic cell line THP1, PARD3 was revealed to contribute to cell migration in response to gradients of inflammatory chemokines^36^. Further, the PARD3-PARD6-aPKC complex has been shown to be downregulated by TNF-α signaling in epithelial cells and in vivo during intestinal inflammation^37^. Therefore, the rare variants in *PARD3* detected here may have a role in acute inflammation via abnormal functioning of tight junctions in SJS/TEN with SOC patients.

We also confirmed the known associations of rs897693 (OR = 4.39, *p* = 2.74 × 10^−6^) and rs4917014 (OR = 0.54, *p* = 2.08 × 10^−5^) associated with differential splicing isoforms of *IKZF1*^14^. We previously performed a meta-analysis of several ethnic groups (not only Japanese, but also Korean, Indian, and Brazilian) and reported that the *IKZF1* gene, especially rs4917014 SNP (OR = 0.5, *p* = 8.46 × 10^−11^)^14^, had a significant genome-wide association with CM-SJS/TEN with SOC. In addition, we recently found significant association between CM-SJS/TEN with SOC and *IKZF1* gene including rs4917014 SNP in Thai population (in submission). We suggest that *IKZF1* might be a universal marker for susceptibility to CM-SJS/TEN with SOC^14^. Our functional analysis of SNPs of the *IKZF1* gene including rs4917014 revealed that the ratio of the splicing isoforms Ik2/Ik1 could be affected by *IKZF1* SNPs and we found that the quantity of Ik2 isoform, which might dominant-negative, is increased in disease-protective genotypes of *IKZF1* (rs4917014 G/G)^14^, suggesting that the function of Ikaros, the protein of *IKZF1*, is enhanced in CM-SJS/TEN with SOC^14^. Moreover, we produced K5-Ikzf1-EGFP transgenic (Ikzf1-Tg) mice by introducing the Ik1 isoform into cells expressing keratin 5, which was expressed in epithelial tissues such as the epidermis and conjunctiva and found that mucocutaneous inflammation was exacerbated in Ikzf1-Tg mice, suggesting that *IKZF1* could play a critical role in maintaining mucocutaneous homeostasis^38^.

We previously performed HLA analysis of CM-SJS/TEN with SOC and reported that in Japanese, HLA-A*02:06 and HLA-B*44:03 were significantly associated with CM-SJS/TEN with SOC^12^, and these associations were not observed in Japanese cases of CM-SJS/TEN without SOC and cold medicine-unrelated (other medicine-related) SJS/TEN with SOC^12^. Therefore, there might be different genetic predisposition between CM-SJS/TEN with SOC and cold medicine-unrelated (other medicine-related) SJS/TEN with SOC.

Moreover, we also reported that PGE_2_ acts at EP_3_ in epidermis and mucosal epithelium and negative regulates mucocutaneous inflammation. CM such as acetaminophen, NSAIDs, and multi-ingredient cold medications similarly down-regulate PGE_2_ which suppress mucocutaneous inflammation. We also suspect that not only CM but also some viral or microbial infections might be important to develop CM-SJS/TEN with SOC. When individuals with a genetic background containing SJS/TEN with SOC susceptibility factors are infected by some viral or microbial infection, they develop abnormal immune responses, and then the administration of cold medicine can down-regulate PGE_2_ inflammatory suppressing mechanism and might augment abnormal immune response resulting in the induction of SJS/TEN with SOC. In contrast, individuals with no genetic susceptibility factors develop a normal immune response upon microbial infection, and the administration of cold medicine has no effect outside of its normal function^36^.

To the best of our knowledge, this is the first study on CM-SJS/TEN with SOC that employed the WGS approach. This enabled extensive discovery of variants, including common and rare SNVs and a variety of SVs. Genome-wide association testing for these variants revealed the regulatory role of common variants, as well as the contribution of rare and structural variants in the development of CM-SJS/TEN with SOC. This study has shed light on the genomic architecture of immune-mediated complex disease and will hopefully be a foundation for more such studies in the future.

## Methods

### Study subjects

In total, 133 patients with CM-SJS/TEN with SOC and 418 healthy volunteers were enrolled in this study. All participants are Japanese descent. DNA sample from 133 patients and all control samples have collected in our previous study^15^. The protocol of the study was approved by the institutional review board at Kyoto Prefectural University of Medicine and the University of Tokyo. Adult patients provided written informed consent, and if the patients were minors (<20 years old), parental consent was obtained. Diagnosis of SJS/TEN by the ophthalmologists was based on a confirmed history of acute onset of high fever, serious mucocutaneous illness with skin eruptions, and involvement of at least two mucosal sites including the ocular surface^39^. We defined patients with SOC as those who manifested pseudomembranes and epithelial defects on the ocular surface (cornea and/or conjunctiva) in the acute stage^40^, and as patients with ocular sequelae such as severe dry eye, trichiasis, symblepharon, and conjunctival invasion into the cornea in the chronic stage^41^.

We have classified the patients who had taken cold medicines such as NSAIDs and multi-ingredient cold medications for a few ~ several days before the disease onset for common-cold symptoms as CM-SJS/TEN. The specific drugs they used were not named by all patients^15^. In Japan, doctors in hospital usually prescribed cold medicine such as NSAIDs and acetaminophen with some antibiotics. However, we previously reported that the use of cold medicine such as NSAIDs and cold-remedies was significantly associated with SJS/TEN with SOC, while the use of antibiotics was not associated^6^.

### Sample preparation and WGS

DNA library preparation and WGS were performed by a contracted laboratory (Toshiba Inc., Tokyo, Japan). DNA libraries were prepared using the TruSeq^®^ DNA PCR-Free Sample Prep Kit (Illumina, San Diego, CA), according to the manufacturer’s instructions. WGS was performed using the Illumina HiSeqX (Illumina, San Diego, CA) with 150-bp paired-end reads. The resultant fastq-formatted raw sequence reads were then shipped to our laboratory.

### WGS analysis

Sequence reads were aligned to hg19 reference sequences after some modifications (tommo_hg19_v2.fa, https://ijgvd.megabank.tohoku.ac.jp/download/tommo_hg19_v2/) by BWA-mem (ver. 0.7.5a-r405)^42^. The duplicate reads were marked using PicardTools (version 1.93) (https://broadinstitute.github.io/picard/). The BAM files were used as input for the discovery and genotyping of autosomal variants described below.

### Discovery and genotyping of polymorphisms

Discovery of SNVs and short INDELs was performed using the HaplotypeCaller implemented in GATK3.8, according to the recommended protocol^43^. Genotype calling was jointly carried out by GenotypeGVCFs. Variant quality score recalibration (VQSR) was applied to the raw genotype call set using the HapMap and International 1000 Genomes Omni2.5 sites as true datasets, the high-confidence SNPs of 1000 G sites as training datasets, and the dbSNP138 sites as known datasets. All variants that passed the 99.9% sensitivity filter were used for subsequent analyses.

Discovery of large deletions (DEL), duplications (DUP), and inversions (INV) was performed using LUMPY v0.2.3^44^, followed by genotype calling using SVTyper v0.6.1^45^. These protocols were performed using the Smoove pipeline v0.2.1, according to author’s instructions (https://github.com/brentp/smoove). Unclassified break-end (BND) loci, SVs with low mean Smoove Het Quality (MHSQ < 3), or SVs spanning longer than 100 kb were filtered out.

Discovery and genotyping of MEI were performed using MELT version 2.1.4^46^, according to the author’s instructions. The filtering criteria provided by MELT were applies, and loci without PASS in the FILTER field of the VCF file were excluded from downstream analyses.

STR variants were analyzed using HipSTR v0.6.1^47^ using the reference files of STR regions provided by the author. PCR stutter models and STR alleles were estimated without external references. STR loci were filtered using the filter_haploid_vcf.py program bundled with HipSTR with options (--min-call-qual 0.9 --max-call-flank-indel 0.15 --max-call-stutter 0.15).

### Association test for single variants

Association tests of case/control samples were conducted for SNVs, short INDELs, and other SVs. The following filters were applied before the association tests: call rate < 95%, minor allele frequency (MAF) < 1% in cases and controls, and multi-allelic loci except for STRs, low-complexity regions, or Hardy-Weinberg equilibrium (HWE) *p* value < 1 × 10^−5^. For STR multiallelic loci, we filtered out loci in which the frequency of the most common allele was larger than 99%, instead of the MAF < 1% criterion. Statistical association was tested under the null model in which there are no difference in the allele count between case and controls for each variant. For biallelic variants (SNVs, short INDELs, DUPs, DELs, INVs, and MEIs), chi-squared tests with 2 × 2 contingency tables were performed using plink1.9^48^. For STR loci, we tested the independence of allele counts between the case and control samples using chi-squared tests with 2 × *n* contingency tables, where n is the number of alleles in a locus calculated by the in-house program.

### Association test for rare variants

EPACTS 3.2.6 (https://github.com/statgen/EPACTS) was used for gene-based tests. Variants with call rates < 97%, MAF > 5%, or HWE *p* value < 1 × 10^−5^ were excluded from this analysis. We evaluated the cumulative effect of rare variants that affect the protein peptide (non-synonymous or stop gain) or RNA splicing (splice site) (in which the variant impact was flagged as “HIGH” or “MODERATE” by the SNPeff program^49^) with the hg19 dataset using Wald’s test and optimal sequence kernel association test (SKAT-O)^50^. These high-impact variants were grouped by gene.

### *eQTL* analysis

Correlations between all SNPs in the human genome and the expression levels of all genes expressed in whole blood were examined using the GTEx portal database (http://gtexportal.org/home/)^19^.

### Reporting summary

Further information on research design is available in the Nature Research Reporting Summary linked to this article.

## Supplementary information

Supplementary Information

Supplementary Data

Reporting Summary

## Data Availability

The summary statistics of the current study are available in the database of NBDC human database; Accession: hum0029.v2.var.v1. All other relevant data are available from the corresponding author (K.T.) upon request.

## References

[1] Yetiv JZ, Bianchine JR, Owen JA (1980). Etiologic factors of the Stevens-Johnson syndrome. South. Med. J..

[2] White KD (2018). SJS/TEN 2017: building multidisciplinary networks to drive science and translation. J. allergy Clin. Immunol. Pract..

[3] Yamane Y, Aihara M, Ikezawa Z (2007). Analysis of Stevens-Johnson syndrome and toxic epidermal necrolysis in Japan from 2000 to 2006. Allergol. Int..

[4] Chan HL (1990). The incidence of erythema multiforme, Stevens-Johnson syndrome, and toxic epidermal necrolysis. A population-based study with particular reference to reactions caused by drugs among outpatients. Arch. Dermatol..

[5] Sotozono C (2015). Predictive factors associated with acute ocular involvement in Stevens-Johnson syndrome and toxic epidermal necrolysis. Am. J. Ophthalmol..

[6] Chung W-H (2004). A marker for Stevens–Johnson syndrome. Nature.

[7] Ozeki T (2011). Genome-wide association study identifies HLA-A*3101 allele as a genetic risk factor for carbamazepine-induced cutaneous adverse drug reactions in Japanese population. Hum. Mol. Genet..

[8] McCormack M (2011). HLA-A*3101 and carbamazepine-induced hypersensitivity reactions in Europeans. N. Engl. J. Med..

[9] Hung S-I (2005). HLA-B*5801 allele as a genetic marker for severe cutaneous adverse reactions caused by allopurinol. Proc. Natl Acad. Sci..

[10] Lonjou C (2008). A European study of HLA-B in Stevens-Johnson syndrome and toxic epidermal necrolysis related to five high-risk drugs. Pharmacogenet. Genomics.

[11] Tohkin M (2013). A whole-genome association study of major determinants for allopurinol-related Stevens–Johnson syndrome and toxic epidermal necrolysis in Japanese patients. Pharmacogenomics J..

[12] Ueta M (2015). Independent strong association of HLA-A*02:06 and HLA-B*44:03 with cold medicine-related Stevens-Johnson syndrome with severe mucosal involvement. Sci. Rep..

[13] Nakatani K (2019). Identification of HLA-A*02:06:01 as the primary disease susceptibility HLA allele in cold medicine-related Stevens-Johnson syndrome with severe ocular complications by high-resolution NGS-based HLA typing. Sci. Rep..

[14] Ueta M (2015). IKZF1, a new susceptibility gene for cold medicine–related Stevens-Johnson syndrome/toxic epidermal necrolysis with severe mucosal involvement. J. Allergy Clin. Immunol..

[15] Ueta M (2017). Genome-wide association study using the ethnicity-specific Japonica array: identification of new susceptibility loci for cold medicine-related Stevens–Johnson syndrome with severe ocular complications. J. Hum. Genet..

[16] Kawai Y (2015). Japonica array: improved genotype imputation by designing a population-specific SNP array with 1070 Japanese individuals. J. Hum. Genet..

[17] Sudmant PH (2015). An integrated map of structural variation in 2504 human genomes. Nature.

[18] Chiang C (2017). The impact of structural variation on human gene expression. Nat. Genet..

[19] Lonsdale J (2013). The Genotype-Tissue Expression (GTEx) project. Nat. Genet..

[20] Apps R (2013). Influence of HLA-C expression level on HIV control. Science.

[21] Ramsuran V (2015). Epigenetic regulation of differential HLA-A allelic expression levels. Hum. Mol. Genet..

[22] Hitomi Y (2019). NFKB1 and MANBA confer disease susceptibility to primary biliary cholangitis via independent putative primary functional variants. Cell. Mol. Gastroenterol. Hepatol..

[23] Peng C (2007). BRD7 suppresses the growth of Nasopharyngeal Carcinoma cells (HNE1) through negatively regulating β-catenin and ERK pathways. Mol. Cell. Biochem..

[24] Zhou M (2006). BRD2 is one of BRD7-interacting proteins and its over-expression could initiate apoptosis. Mol. Cell. Biochem..

[25] Xu K (2016). Integrating ChIP-sequencing and digital gene expression profiling to identify BRD7 downstream genes and construct their regulating network. Mol. Cell. Biochem..

[26] Zhang Q (2016). Bromodomain containing protein represses the Ras/Raf/MEK/ERK pathway to attenuate human hepatoma cell proliferation during HCV infection. Cancer Lett..

[27] Zhao R (2017). BRD7 plays an anti-inflammatory role during early acute inflammation by inhibiting activation of the NF-кB signaling pathway. Cell. Mol. Immunol..

[28] Chalmers IJ, Höfler H, Atkinson MJ (1999). Mapping of a cadherin gene cluster to a region of chromosome 5 subject to frequent allelic loss in carcinoma. Genomics.

[29] Keh SM (2011). The menthol and cold sensation receptor TRPM8 in normal human nasal mucosa and rhinitis. Rhinology.

[30] Kono T (2012). Oxaliplatin-induced neurotoxicity involves TRPM8 in the mechanism of acute hypersensitivity to cold sensation. Brain Behav..

[31] Park N-H, Na Y-J, Choi H-T, Cho J-C, Lee H-K (2013). Activation of transient receptor potential melastatin 8 reduces ultraviolet B-induced prostaglandin E2 production in keratinocytes. J. Dermatol..

[32] Ueta M (2011). Receptor subtype EP3 expression in human conjunctival epithelium and its changes in various ocular surface disorders. PLoS One.

[33] Johansson A, Driessens M, Aspenström P (2000). The mammalian homologue of the Caenorhabditis elegans polarity protein PAR-6 is a binding partner for the Rho GTPases Cdc42 and Rac1. J. Cell Sci..

[34] Suzuki A (2001). Atypical protein kinase C is involved in the evolutionarily conserved par protein complex and plays a critical role in establishing epithelia-specific junctional structures. J. Cell Biol..

[35] Brinkmann BF (2016). VE-cadherin interacts with cell polarity protein Pals1 to regulate vascular lumen formation. Mol. Biol. Cell.

[36] Tamehiro N (2009). Cell polarity factor Par3 Binds SPTLC1 and modulates monocyte serine palmitoyltransferase activity and chemotaxis. J. Biol. Chem..

[37] Mashukova A, Wald FA, Salas PJ (2011). Tumor necrosis factor alpha and inflammation disrupt the polarity complex in intestinal epithelial cells by a posttranslational mechanism. Mol. Cell. Biol..

[38] Ueta M (2018). Mucocutaneous inflammation in the Ikaros Family Zinc Finger 1-keratin 5–specific transgenic mice. Allergy.

[39] Ueta M (2020). Stevens-Johnson syndrome/toxic epidermal necrolysis with severe ocular complications. Expert Rev. Clin. Immunol..

[40] Sotozono C (2009). Diagnosis and treatment of Stevens-Johnson syndrome and toxic epidermal necrolysis with ocular complications. Ophthalmology.

[41] Sotozono C (2007). New grading system for the evaluation of chronic ocular manifestations in patients with Stevens–Johnson syndrome. Ophthalmology.

[42] Li H, Durbin R (2009). Fast and accurate short read alignment with Burrows-Wheeler transform. Bioinformatics.

[43] Van der Auwera, G. A. et al. From fastQ data to high-confidence variant calls: the genome analysis toolkit best practices pipeline. in *Current Protocols in Bioinformatics*. (John Wiley & Sons, Inc., 2013). 10.1002/0471250953.bi1110s43 10.1002/0471250953.bi1110s43 PMC4243306 25431634

[44] Layer RM, Chiang C, Quinlan AR, Hall IM (2014). LUMPY: a probabilistic framework for structural variant discovery. Genome Biol..

[45] Chiang C (2015). SpeedSeq: ultra-fast personal genome analysis and interpretation. Nat. Methods.

[46] Gardner EJ (2017). The Mobile Element Locator Tool (MELT): population-scale mobile element discovery and biology. Genome Res..

[47] Willems T (2017). Genome-wide profiling of heritable and de novo STR variations. Nat. Methods.

[48] Chang CC (2015). Second-generation PLINK: rising to the challenge of larger and richer datasets. Gigascience.

[49] Cingolani P (2012). A program for annotating and predicting the effects of single nucleotide polymorphisms, SnpEff. Fly. (Austin)..

[50] Lee S, Wu MC, Lin X (2012). Optimal tests for rare variant effects in sequencing association studies. Biostatistics.

