# Supplementary material for: Mapping of susceptible variants for cold medicine-related Stevens–Johnson syndrome by whole-genome resequencing
Source: NPJ Genom Med. 2021 Feb 11;6:9. doi: 10.1038/s41525-021-00171-2 (PMC7878485; doi:10.1038/s41525-021-00171-2)
Supplement: Supplementary file 1 — Supplementary Information [file 41525_2021_171_MOESM1_ESM.pdf]

a

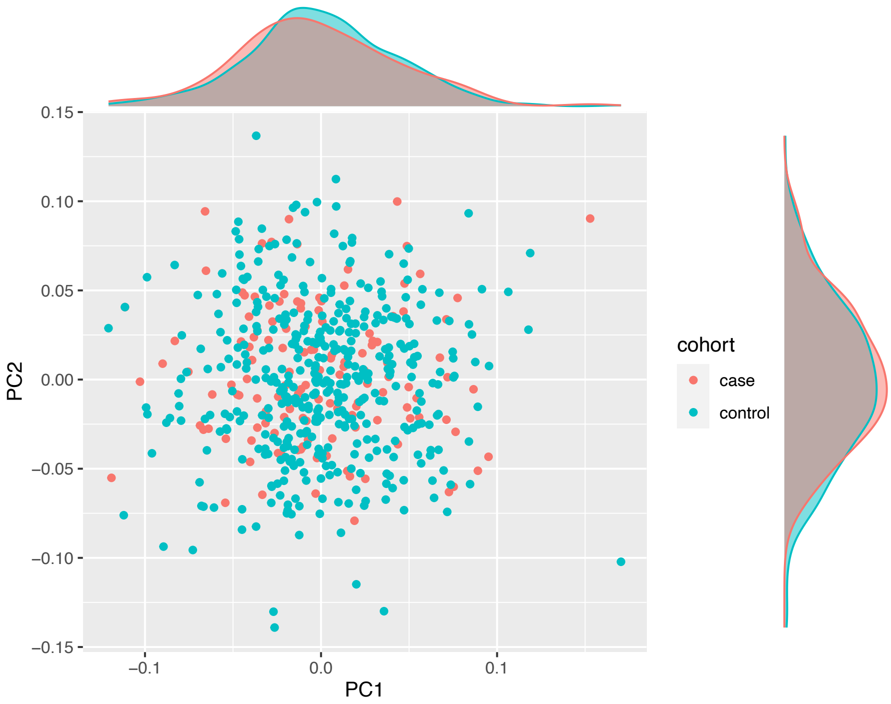

b

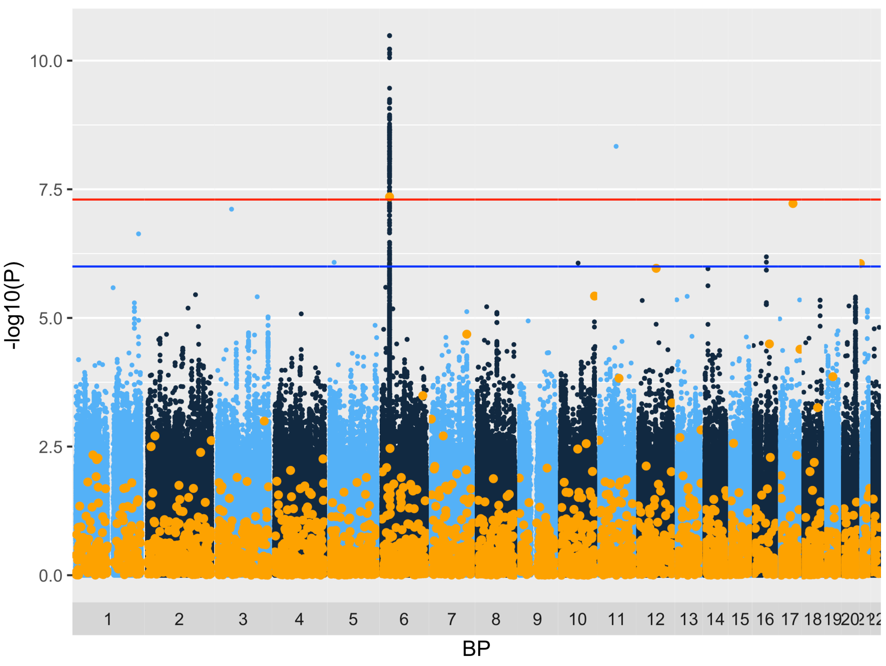

Supplementary Figure 1 **a**. PCA plot for 133 SJS patients (case) and 418 controls. **b**. Genome wide association analysis of logistic regression adjusted by sex and the first four principal components as covariates.

```
                       chr5:21895127
                            ↓
5’-AAATAAATTG AAAAAAAAAAAAA--- GACTAGTT-3’  12
5’-AAATAAATTG AAAAAAAAAAAAAA-- GACTAGTT-3’  13(hg19)
5’-AAATAAATTG AAAAAAAAAAAAAAA- GACTAGTT-3’  14
5’-AAATAAATTG AAAAAAAAAAAAAAAA GACTAGTT-3’  15
```

Supplementary Figure 2. STR locus representing four alleles that have 12, 13, 14, and 15 adenine (A) repeats in the intronic region of the cadherin 12 (*CDH12*) gene.

Supplementary Table 1. Summary of variants discovered in this study

Type of variant	Discovery tool	Number of variants before filtering	Number of variants after filtering	Number of variants per individual (case) (mean ± sd)	Number of variants per individual (control) (mean ± sd)
SNV	GATK HaplotypeCaller	24,203,167	18,824,284	3,153,437 ± 13,808	3,156,271 ± 20,512
Indel	GATK HaplotypeCaller	4,200,793	1,702,192	287,098 ± 1,289	287,621 ± 1,808
DEL	LUMPY	20,199	2,065	1,024 ± 19.5	1,006 ± 19.5
DUP	LUMPY	5,605	273	154 ± 3.8	155 ± 4.4
INV	LUMPY	870	55	30.5 ± 2.8	29.1 ± 3.1
MEI	MELT	15,379	7,821	1,102 ± 22.1	1101 ± 22.8
STR	HipSTR	1,471,980	670,775	248,832 ± 2,573	250,368 ± 1,783

Supplementary Data 1. The variants showing the association with the development of CM-SJS/TEN with p-value < 1.0 x 10^{-6}.

Supplementary Data 2. The missense and stop-gained variants detected in *TRPM8* gene.
