# Supplementary material for: Mapping of susceptible variants for cold medicine-related Stevens–Johnson syndrome by whole-genome resequencing
Source: NPJ Genom Med. 2021 Feb 11;6:9. doi: 10.1038/s41525-021-00171-2 (PMC7878485; doi:10.1038/s41525-021-00171-2)
Supplement: Supplementary file 3 — Reporting Summary [file 41525_2021_171_MOESM3_ESM.pdf]

nature research

Corresponding author(s): Mayumi Ueta and Katsushi Tokunaga

Last updated by author(s): Dec 18, 2020

Reporting Summary

Nature Research wishes to improve the reproducibility of the work that we publish. This form provides structure for consistency and transparency in reporting. For further information on Nature Research policies, see our Editorial Policies and the Editorial Policy Checklist.

Statistics

For all statistical analyses, confirm that the following items are present in the figure legend, table legend, main text, or Methods section.

n/a	Confirmed	
☐	☒	The exact sample size (n) for each experimental group/condition, given as a discrete number and unit of measurement
☐	☒	A statement on whether measurements were taken from distinct samples or whether the same sample was measured repeatedly
☐	☒	The statistical test(s) used AND whether they are one- or two-sided<br>*Only common tests should be described solely by name; describe more complex techniques in the Methods section.*
☐	☒	A description of all covariates tested
☐	☒	A description of any assumptions or corrections, such as tests of normality and adjustment for multiple comparisons
☐	☒	A full description of the statistical parameters including central tendency (e.g. means) or other basic estimates (e.g. regression coefficient) AND variation (e.g. standard deviation) or associated estimates of uncertainty (e.g. confidence intervals)
☐	☒	For null hypothesis testing, the test statistic (e.g. F, t, r) with confidence intervals, effect sizes, degrees of freedom and P value noted<br>*Give P values as exact values whenever suitable.*
☒	☐	For Bayesian analysis, information on the choice of priors and Markov chain Monte Carlo settings
☒	☐	For hierarchical and complex designs, identification of the appropriate level for tests and full reporting of outcomes
☐	☒	Estimates of effect sizes (e.g. Cohen's d, Pearson's r), indicating how they were calculated

Our web collection on statistics for biologists contains articles on many of the points above.

Software and code

Policy information about availability of computer code

Data collection	No software was used for data collection.
Data analysis	BWA-mem (ver. 0.7.5a-r405), PicardTools (version 1.93), GATK3.8, LUMPY v0.2.3, SVTyper v0.6.1, Smoove pipeline v0.2.1, MELT version 2.1.4, HipSTR v0.6.1, plink1.9, r-language 4.0

For manuscripts utilizing custom algorithms or software that are central to the research but not yet described in published literature, software must be made available to editors and reviewers. We strongly encourage code deposition in a community repository (e.g. GitHub). See the Nature Research guidelines for submitting code & software for further information.

Data

Policy information about availability of data

All manuscripts must include a data availability statement. This statement should provide the following information, where applicable:

- Accession codes, unique identifiers, or web links for publicly available datasets
- A list of figures that have associated raw data
- A description of any restrictions on data availability

Summary statistics from genome-wide association tests are available in the database of MGeND; Accession: hum0029.v2.var.v1. All other relevant data are available from the corresponding author (K.T.) upon request.

Field-specific reporting

Please select the one below that is the best fit for your research. If you are not sure, read the appropriate sections before making your selection.

☒ Life sciences ☐ Behavioural & social sciences ☐ Ecological, evolutionary & environmental sciences

For a reference copy of the document with all sections, see nature.com/documents/nr-reporting-summary-flat.pdf

Life sciences study design

All studies must disclose on these points even when the disclosure is negative.

Sample size	All patients who were diagonalized to be CM-SJS/TEN and provided the written consent and were enrolled.
Data exclusions	We applied the standard quality control procedures for variant calling from NGS data and association analysis. The detailed procedures and criteria were described in Material and Methods section.
Replication	Replication of GWAS was not conducted due to the limited sample availability. However, the major results of single variant association analysis were consistent with out previous study where the different approach (SNP array + genotype imputation) was applied. This limitation was discussed in the manuscript.
Randomization	Genome-wide association tests were conducted adjusting sex and four principal components.
Blinding	Investigators were blinded to patients' genomic profile during data analysis

Reporting for specific materials, systems and methods

We require information from authors about some types of materials, experimental systems and methods used in many studies. Here, indicate whether each material, system or method listed is relevant to your study. If you are not sure if a list item applies to your research, read the appropriate section before selecting a response.

Materials & experimental systems

n/a	Involved in the study	
☒	☐	Antibodies
☒	☐	Eukaryotic cell lines
☒	☐	Palaeontology and archaeology
☒	☐	Animals and other organisms
☐	☒	Human research participants
☒	☐	Clinical data
☒	☐	Dual use research of concern

Methods

n/a	Involved in the study	
☒	☐	ChIP-seq
☒	☐	Flow cytometry
☒	☐	MRI-based neuroimaging

Human research participants

Policy information about studies involving human research participants

Population characteristics	133 Japanese individuals with CM-SJS/TEN with SOC and 418 healthy Japanese volunteers
Recruitment	Patients were recruited from Kyoto Prefectural University of Medicine Hospital.
Ethics oversight	Kyoto Prefectural University of Medicine and the University of Tokyo

Note that full information on the approval of the study protocol must also be provided in the manuscript.
